# How staying in a gymnasium affects sleep and bed climate in children

**DOI:** 10.1186/s40101-023-00350-3

**Published:** 2024-01-02

**Authors:** Kazue Okamoto-Mizuno, Koh Mizuno

**Affiliations:** 1 Faculty of Human Ecology, Wayo Women’s University, 2-3-1 Konodai, Ichikawa, Chiba, 272-8533 Japan; 2 Faculty of Education, Tohoku Fukushi University, 6-149-1 Kunimigaoka, Aoba, Sendai, Miyagi 989-3201 Japan

**Keywords:** Bed climate, Sleep, Simulated shelter, Gymnasium, Children

## Abstract

**Background:**

We investigated the relationship between sleep, ambient climate, and bed climate in school-aged children during a one-night stay in a simulated shelter in a gymnasium to demonstrate the effect of ambient climate, and bed climate on sleep.

**Methods:**

We obtained measurements during a one-night stay in a school gymnasium (C), days before C (BC), on the day after (A1), and on the second day after C (A2) in 13 healthy school-aged children during summer. Sleep was evaluated using an actigraph, and the temperature and humidity of the bedrooms in the participants’ homes and in the gymnasium were monitored for 3 days before and after C. The bed climate of the chest and foot areas was measured for two nights before and after C. The participants were asked to report on their subjective sleep estimations and thermal sensations two nights before and after C.

**Results:**

The ambient temperature in C was significantly higher than that in BC at the initial 180 min, while it significantly decreased compared to A1 and A2 in the last 100 min. The ambient humidity was significantly higher in both C and BC compared to A1 and A2. The sleep efficiency index decreased significantly in C (43±4.8%) compared to other conditions. Sleep time was significantly shorter in C than in other conditions during the initial 195 min. The increase in the bed climate temperature of the chest area in C was significantly delayed compared to that in the other conditions, around the initial 30 min after the lights were off. Subjective estimation revealed that in C, 85% of the participants were out of their sleeping bag at sleep onset, and their sleep was disturbed by heat (77%).

**Conclusions:**

Our study revealed that the disturbed sleep patterns observed with children in a simulated shelter may be related to a delayed increase in bed climate temperature in the chest area. This delayed increase could be related to the children not entering the sleeping bag and a delayed chest skin temperature increase during the sleep onset period.

## Background

In the case of impactful disasters such as the Great East Japan Earthquake of 2011, many shelters were established in a park and in school gymnasiums [1]. Previous studies indicate that staying in shelters after a disaster is accompanied by sleep disturbances due to various factors. In the elderly who evacuated to a school gymnasium during the Great East Japan Earthquake, severe sleep disturbances were identified using actigraphic sleep measures. These disturbances were due to cold sensations from the floor, anxiety, and aftershocks [2]. In a survey of 250 evacuees who stayed in a gymnasium for 1.5 months after the Great East Japan Earthquake, the most frequently noted reasons for sleep disturbances were noise, a sensation of cold coming from the floor, and lighting [3]. In this survey, a cold sensation from the floor was claimed despite an ambient temperature of 20°C. In our previous study conducted in the summertime, during a one-night stay in a school gymnasium with 109 people, children slept for a shorter period of time, while adults experienced no effect [4]. Sleep disturbance factors in children included noise, lighting, uncomfortable sleeping bags, and heat, while the stiffness of the floor, uncomfortable pillows, noise, and cold were factors for adults. In our another prior study in the summertime, children’s sleep was severely disturbed during a one-night stay in a gymnasium, mainly due to heat, even at an ambient temperature of around 26°C [5]. These previous studies suggest that environmental factors, especially thermal discomfort, are among the most important elements affecting sleep in shelters. Thermal discomfort during sleep is related to both ambient climate and bedding conditions because most shelters in Japan lack air conditioning, sufficient space, adequate bedding, and portable beds, which cause evacuees to sleep directly on the solid floor.

The thermal environment is one of the most critical environmental issues involving sleep. The temperature and humidity of the microclimate between humans and clothing (microclimate) and bed covers (bed climate) also play crucial roles in creating a warm and dry bed climate to support sleep [6]. The bed climate temperature and relative humidity (RH) in the waist area are generally maintained around 32–34°C, 40–60% RH during normal sleep [7, 8], which is in accordance with the range of comfort bed climate [9]. Furthermore, bed climate humidity higher than 70% is related to sleep disturbance under high ambient temperatures in adults [9]. In school-aged children, the bed climate temperature of the foot area in summer and fall is maintained at 28–30°C, 50–70% RH when sleep is in the normal range [10]. However, to the best of our knowledge, it is still unknown whether the bed climate of school-aged children is related to sleep. Since sleep disturbance factors in children staying in the gymnasium include hot environments and uncomfortable sleeping bags, it is of great interest to investigate the relationship between sleep and bed climate in children sleeping in a shelter. In our previous study, ambient climate and sleep were measured, and subjective assessments were asked to 20 school-aged children during a one-night stay in the gymnasium [5]. In the same study, we measured bed climate in 15 out of these 20 children. However, since the previous study focused on various ambient environment factors and sleep, including naps, the results of bed climate as well as the time course of ambient climate were not considered. We aimed to investigate the relationship among sleep, ambient climate, and bed climate in school-aged children staying in a simulated shelter in a school gymnasium. This information may shed light on the thermal environmental factors that affect sleep in a shelter and contribute to practical applications for disaster prevention in the future.

## Material and methods

### Participants

The methods for screening the participants and the details of the participants were the same as those in our previous study [4, 5, 11]. Among 20 participants in our previous study, 15 participants who were in the fifth and sixth grades in Japanese elementary school were selected for bed climate measurements together with sleep parameters and subjective assessments. We excluded two participants from the final analysis because of the termination of bed climate measurements due to body movements. Finally, we analyzed 13 (6 males and 7 females) participants. The means and standard deviations (SDs) of the children’s age and physical characteristics were 11.4±0.7 years (age), 152.2±4.9 cm (height), 32.5±7.0 kg (weight), and a Lohrel index of 109.6±9.2. The study’s protocol was informed to the participants and their parents, prior to giving their written consent. This study was approved by the Ethics Committee of Tohoku Fukushi University.

### Procedure

We performed the study during a one-night stay in a school gymnasium in summertime; the event was organized at an elementary school (summer camp). In total, 174 people slept in the gymnasium (123 children, 45 parents, and six teachers).

The objectives and details of the summer camp have been described in a previous study of ours [5]. The participants were asked to wear a wrist actigraph (Micromini, AMI) on their non-dominant wrist for 7 consecutive days; from 3 days prior to 3 days after the school camp. Measurements were then recorded from Wednesday to Tuesday. The children attended school until 12:30 on Saturdays, and Sundays and Mondays were holidays. The actigraphs were removed when children attended school on weekdays. Their mothers were asked to record information in a sleep log, as well as instances in which their children removed their actigraph. During the activity monitoring period, we continuously measured the temperature, relative humidity (RH), and lighting in the bedroom at 2-min intervals using a thermistor, hygrometer, and illumination meter (RS-12, Espec Mic Corp). At the school camp, we continuously measured the nocturnal temperature, RH, and lighting of the gymnasium at 1-min intervals using a thermistor, hygrometer, and illumination meter (RS-12, Espec Mic Corp). We continuously measured the noise of the gymnasium at 2-s intervals using four noise sensors (CENTER322, Center Technology Corp.). The floor area of the gymnasium was approximately 1050 m^2^ (approximately 28 m × 37 m). The details of the gymnasium and noise measurement areas have been described in our previous study [5]. We continuously measured the bed climate of the chest and foot area at 1-min intervals for five nights, during the two nights prior to and the two nights after the school camp. The thermistor and hygrometer sensors (RS-12, Espec Mic Corp.) were attached to the center of the chest in the outer layer of the clothing for the chest area and attached to the foot for the foot area. The mothers were asked to attach the sensors to their children starting at least 30 min prior to sleep until their children woke up. Researchers in the gymnasium attached the sensors at the camp night one hour prior to the lights being turned off.

One night prior to and two nights after the school camp, we administered a subjective sleep evaluation. The scales used for subjective sleep evaluation were as follows: sleep onset, +2, easy; +1, slightly easy; 0, neutral; −1, slightly difficult; −2, difficult; overall sleep, +2, good; +1, slightly good; 0, neutral; −1, slightly bad; and −2, bad. To understand the thermal, sweat, and humidity sensations, the participants were provided a questionnaire on how they felt before and after sleep. The scales used for subjective sensations were as follows: thermal sensation, +3, very hot; +2, hot; +1, slightly hot; 0, neutral; −1, slightly cold; −2, cold; −3, very cold; sweat sensation, 0, not sweating; +1, slightly sweating; +2, sweating; +3, very sweating; humid sensation, +2, humid; +1, slightly humid; 0, neutral; −1, slightly dry; and −2, dry). As for the scales to evaluate sleep and subjective sensations, the children were asked to answer one discrete number on each scale. Questionnaires on bedding and clothing were asked, and a digital balance (KD-812, Tanita Corporation) was handed out to mothers to measure the weight of their children’s clothing. On the camp day, the identical sleeping bag and mattress were handed out to the children. The sleeping bag was made of 100% polyester with a 1.1 kg weight and 800×2100 mm in size. The dimensions of the underside mattress were 1800 mm × 600 mm × 8 mm. The participants answered a questionnaire after sleep on how they used the sleeping bag before sleep, during sleep, and upon waking: no usage, using a sleeping bag under the body, and staying inside the sleeping bag. The participants were asked to perform their typical daily activities as usual as possible.

### Data analysis

We re-analyzed the bedroom climate, sleep parameters, clothing weight, and subjective assessments regarding clothing, bedding, and bedroom conditions; subjective sleep evaluation; and thermal and sweat sensation before sleep from our previous study in 13 out of the 20 participants [5]. We scrutinized all data based on days before camp (BC), the night of the camp (C), the day after camp (A1), and 2 days after camp (A2). We determined sleep parameters using an actigram-based sleep-wake identification algorithm. We used the software Action W (AMI) for sleep-wake scoring using Sadeh’s [12, 13] algorithm. Furthermore, time scored as sleep was analyzed every 15 min throughout the night to determine the sleep duration. To test the statistical significance of the data, we used a one-way analysis of variance (ANOVA) (days, BC, C, A1, and A2) for repeated measures to analyze sleep parameters, clothing weight, and subjective sleep estimation. Additionally, a repeated measures two-way ANOVA (days and time) was used to compare temperature, RH, light in the bedroom, sleep duration in each 15-min interval throughout the night, bed climate, and subjective sensations before and after sleep. Because sleep duration differed among the children and conditions, we examined the first 380 min, which is the shortest sleep time observed in A2. We used the Bonferroni method and *t* test for post hoc pairwise comparisons. We set the level of significance at *P*<0.05.

## Results

### Daytime schedule of the summer camp

The daytime schedule of the summer camp has been described in our previous study [5]. In short, children gathered at the school gymnasium at 16:45, slept from 22:00 to 6:00, and left at 8:30 the next day.

### Bedroom climate and environment of the gymnasium

**Fig. 1:**
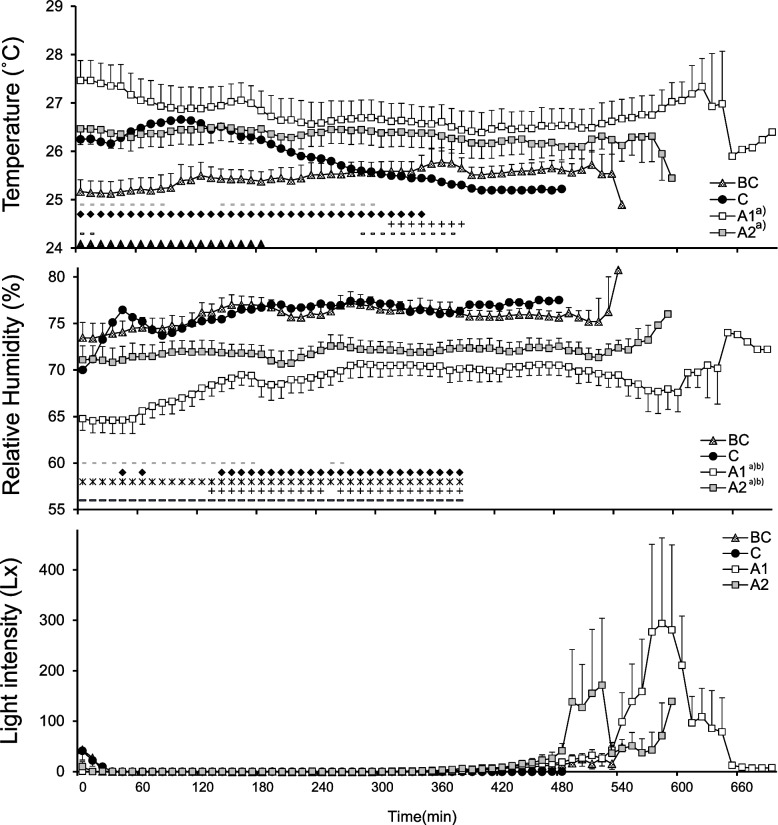
Temperature, relative humidity, and light intensity of the bedroom and gymnasium. ▲ a significant difference (*P*<0.05) between C and BC; **־** a significant difference between C and A1; + a significant difference between C and A2; * a significant difference between BC and A1;♦ a significant difference between BC and A2; **־**a significant difference between C and A1

Figure 1 presents the average temperature and humidity of the bedroom and the light intensity during the time spent in bed. The bedroom temperature differed significantly depending on the condition (*F*_(3, 36)_ = 8.16; *P* < 0.001) and interaction (*F*_(57, 684)_ = 7.14; *P* < 0.001). The bedroom temperature in BC and C was lower than that in A1 and A2. During the initial 180 min, C was significantly higher than BC, while C was significantly lower than A1 and A2 in the last 100 min. The bedroom temperature in C fell from 26.7°C to 25°C, while there were no significant changes in the other conditions. The bedroom RH differed significantly depending on the condition (*F*_(3, 36)_ = 29.6; *P* < 0.001) and interaction (*F*_(57, 684)_ = 2.90; *P* < 0.001). The bedroom RH in A1 and A2 was significantly lower than that in BC and C. During the initial segment of sleep, the bedroom RH in A1 was lower than that in the other conditions, while the RH in A1 and A2 was lower than that in C and BC in the later segment of sleep. The outside mean and maximum temperatures during the day and night were as follows: BC, day, 21.8/23.1°C (mean/maximum) and night, 22.8/25.3°C; C, day, 27.8°C/30.8°C and night, 24.3 °C/27.1°C; A1, day, 28.3°C/31.5°C and night, 24.6 °C/26.1°C; and A2, day, 25.1°C/28,8°C and night, 22.8°C/24.4°C [14]. The outside temperature increased by 6°C during the day and approximately 2°C at night when comparing BC to C. We did not observe any significant differences depending on lighting intensity. The mean noise inside the gymnasium during the light-off period was 44.1±9.4 dB, ranging from 31.5 to 77.5 dB.

The bedding condition at home was a mattress or futon with one covering. The same underside mattress and sleeping bag were used in C (Table 1). No significant difference was observed in the number of layers of mattresses used by the subjects in BC, A1, and A2. Clothing weight significantly differed depending on the condition (*F*_(3, 36)_ = 4.79; *P* < 0.01), with significantly increased weight in BC compared to A2.

**Table 1:**
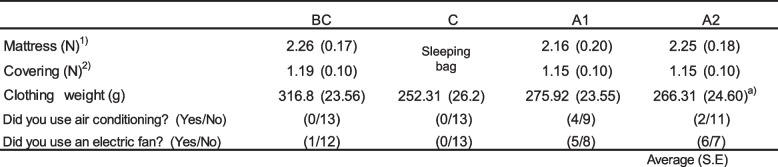
Bedroom and bedding condition during sleep

^a)^a significant difference between BC (P<0.05)

^1)2)^Number of layers of mattress and bed coverings

### Sleep parameters

**Fig. 2:**
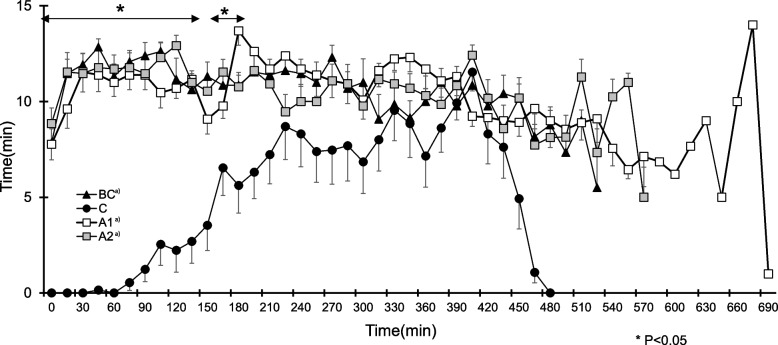
The sleep time during each 15-min period during the night. *P<0.05 a significant difference between C and other conditions

Based on the wrist actigraph findings, we noted significant differences in sleep parameters between the C and the other conditions (Table 2). No significant differences were observed in bedtimes, whereas wake-up times (*F*_(3, 36)_ = 12.11; *P* < 0.001) were significantly earlier in C than those in BC, and later in A1 than those in BC and C. Additionally, the time in bed in A1 was significantly longer than that in C (*F*_(3, 36)_ = 7.35; *P* < 0.01). The total sleep time was significantly shorter in C as compared to all the other conditions (*F*_(3, 36)_ = 46.76; *P* < 0.001). The mean activity (*F*_(3, 36)_ = 95.75; *P*<0.001), wake time (*F*_(3, 36)_ = 69.80; *P* < 0.001), episodes while awake (*F*_(3, 36)_ = 9.55; *P* < 0.001), mean number of episodes while awake (*F*_(3, 36)_ = 12.99; *P* < 0.001), the longest episode of being awake (*F*_(3, 36)_ = 38.43; *P* < 0.001), and sleep latency significantly (*F*_(3, 36)_ = 42.60; *P* < 0.001) increased in C compared to the other conditions. Further, the sleep efficiency index (SEI) was significantly lower in C than in the other conditions (*F*_(3, 36)_ = 72.84; *P* < 0.001). The SEI decreased by approximately half compared to other conditions. Daytime sleep time significantly increased in A1 as compared to all the other conditions (*F*_(3, 36)_ = 12.30; *P* < 0.001). The sleep time during each 15-min period during the night was significantly affected by the condition (*F*_(1.4, 36)_ = 34.72; *P* < 0.0001). The interaction was significant (*F*_(46.2, 555.4)_ = 5.10; *P* < 0.001) (Fig. 2). The sleep time was significantly lower during the first 150 min and 165 to 195 min in condition C compared to the other conditions.

**Table 2:**
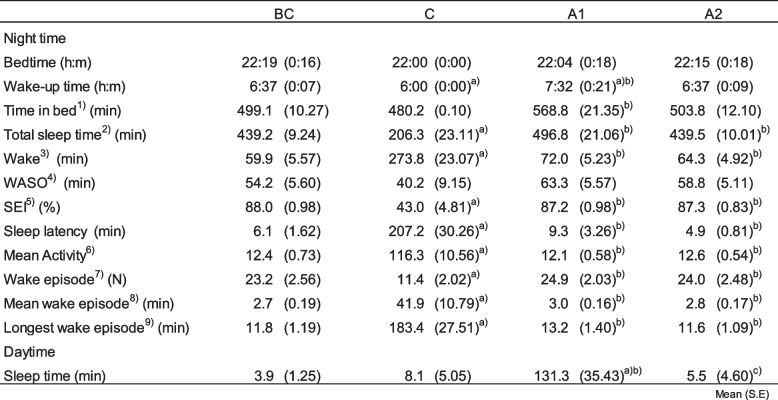
Sleep parameters before and after camp

^1)^Time in bed (TIB), minutes from time when subjects laid down on the bed and lights went off to wake up time; ^2)^TST(total sleep time),total minutes of sleep during time in bed; ^3)^Wake, total minutes of wakefulness during time in bed; ^4)^WASO(Wake after sleep onset), minutes scored as wakefulness from sleep onset to wake up time; ^5)^SEI(sleep efficiency index), SEI=Total sleep time/time in bed; ^6)^Mean activity, the total activity counts measured from actigraph/time in bed (min); ^7)^Wake episode, total number of wake episodes; ^8)^Mean wake episode, wakefulness/total number of wake episode; ^9)^Longest wake episode, longest wake episode during time in bed. ^a)^a significant difference (P,0.05) between BC; ^b)^a significant difference between C; ^c)^a significant difference between A1

### The bed climate of the chest and foot area

**Fig. 3:**
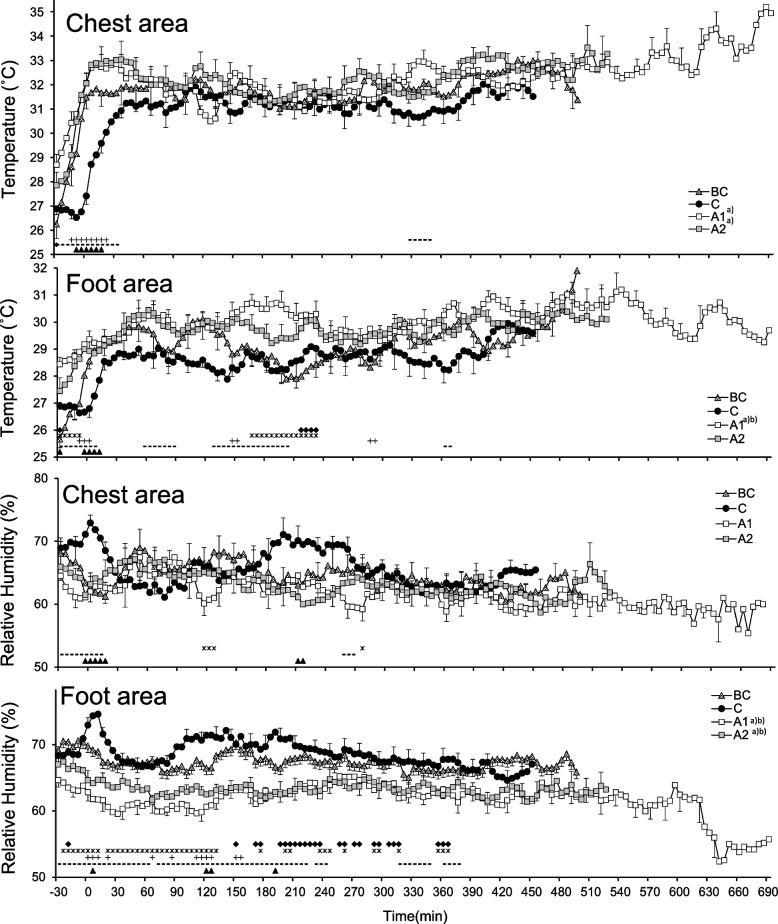
Bed climate of the chest and foot area. ▲ a significant difference (*P*<0.05) between C and BC; **־** a significant difference between C and A1; + a significant difference between C and A2; * a significant difference between BC and A1; ♦ a significant difference between BC and A2

The changes in the bed climate temperature of the chest and foot areas and bed climate humidity of the chest and foot areas are depicted in Fig. 3. The bed climate temperature of the chest area differed significantly depending on the condition (*F*_(3, 36)_ =5.84; *P* < 0.01) and interaction (*F*_(243, 2916)_ = 1.96; *P* < 0.001), which was significantly lower in C than that in A1 and A2. The increase in bed climate temperature in the chest area was significantly delayed in C than in the other conditions during the initial 15 to 30 min of the sleep segment and was significantly lower than that in A1 at 300 to 310 min. The bed climate temperature of the foot area differed significantly depending on the condition (*F*_(3, 36)_ =6.88; *P* < 0.001) and interaction (*F*_(243, 2916)_ =1.29; *P* < 0.001). The bed climate temperature of the foot area in BC and C was significantly lower than that in A1. The temperature increase in the bed foot area was also significantly delayed in C compared to other conditions during the initial 5 to 15 min of sleep. Further, the bed climate temperature of the foot area in BC was significantly lower than that in A1 and A2 before the lights were off. A significantly lower temperature in C than in A2 was observed intermittently from 60 to 210 min, while a significant difference between C and A1 was observed several minutes after the 15 min. There was a significantly lower temperature in BC than in A2 and A1 observed intermittently from 170 to 240 min. The bed climate RH of the chest area differed significantly depending on the interaction (*F*_(243, 2916)_ =2.15; *P* < 0.001). The bed climate RH of the chest area was significantly higher in C as compared to that in A1 and BC during the initial 10 to 15 min of sleep. The bed climate RH of the foot area differed significantly depending on the condition (*F*_(3, 36)_ =30.0; *P* < 0.001) and interaction (*F*_(243, 2916)_ =1.46; *P* < 0.001), which was significantly higher in BC and C as compared to A1 and A2. The bed climate humidity of the foot area was significantly higher in BC and C than in A1 intermittently throughout the night, while it was higher in BC than in A2 during the later sleep segment. No significant differences were observed in the bed climate absolute humidity of the chest and foot areas.

### Subjective assessments

**Fig. 4:**
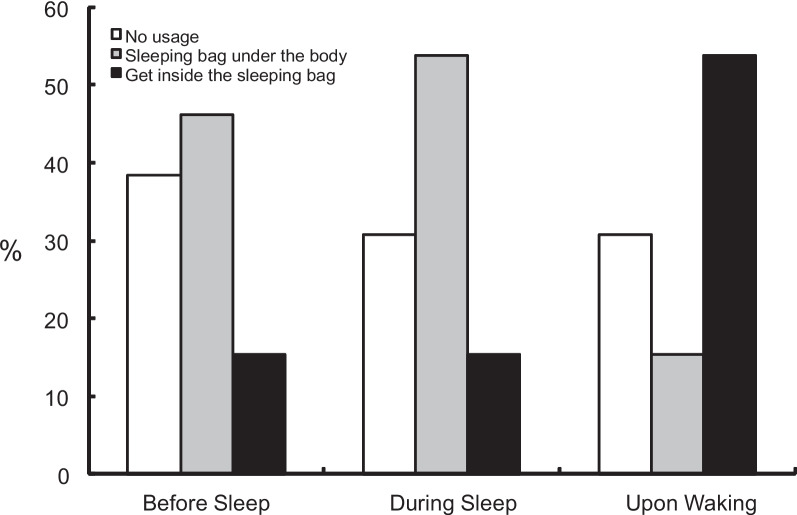
The usage of a sleeping bag during sleep

**Fig. 5:**
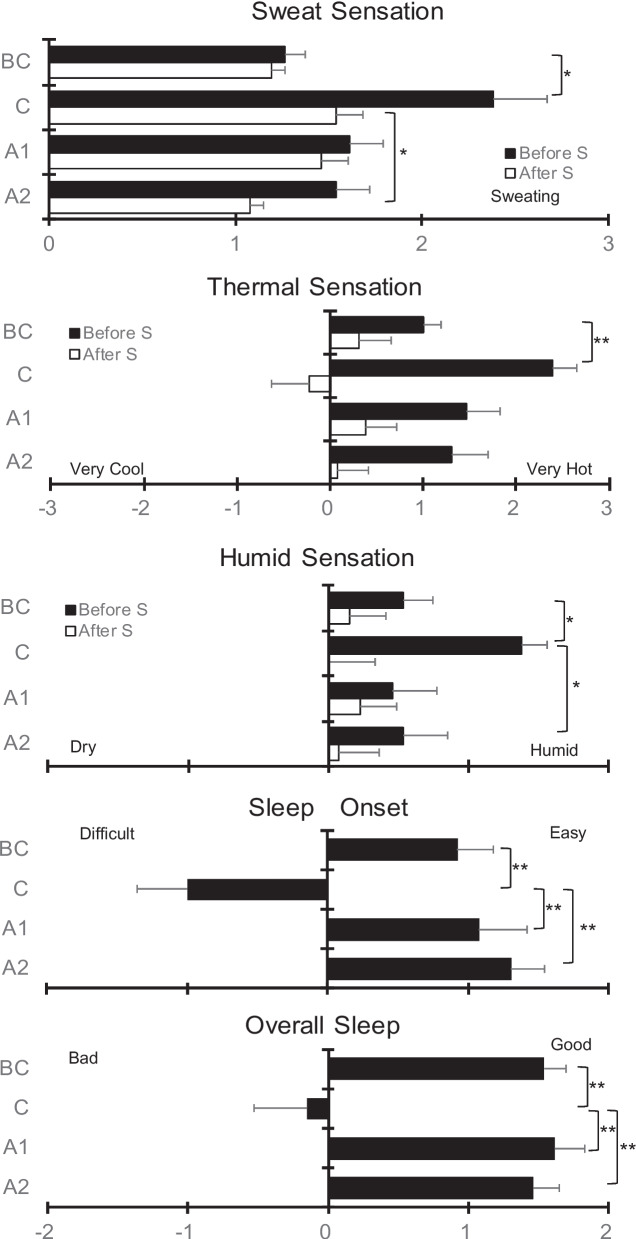
Thermal sensation during sleep and subjective sleep evaluation. *P<0.05, **P<0.01

Regarding the usage of the sleeping bag in C, 15% of participants spent time inside the sleeping bag before going to sleep, and this figure increased to 50% in the later segment of sleep before the wake-up time (Fig. 4). We noted significant differences among the conditions in the subjective overall assessments of sleep (*F*_(3, 36)_ =13.30; *P* < 0.001) and sleep onset (*F*_(3, 36)_ =15.53; *P* < 0.001). The subjective overall assessment of sleep was significantly worse, and sleep onset latency was longer in C than in the other conditions (Fig. 5). Regarding subjective sensations before and after sleep, a significant effect of condition, time, and interaction was observed for sweat (condition, *F*_(3, 36)_ = 4.01; *P* < 0.001; time, *F*_(1,12__)_ =6.36; *P* < 0.001; interaction, *F*_(2.2, 27.3)_ =3.03; *P* < 0.001) and humid sensations (condition, *F*_(3, 36)_ =3.51; *P* < 0.001; time, *F*_(1,12)_ =8.15; *P* < 0.001; interaction, *F*_(3, 36)_ =3.03; *P* < 0.001). Sweat sensations increased in C as compared to that in BC, before sleep, and increased in C as compared to that in A2, after sleep. The humidity before sleep was significantly higher in C than that in BC and A2. Additionally, a significant effect of condition (*F*_(3, 36)_ =14.9; *P* < 0.001) and interaction (*F*_(1.9, 22.8)_ =3.71; *P* < 0.001) was observed for thermal sensations. The thermal sensation before sleep was significantly higher in C than that in BC. The most frequent reason for sleep disturbances during camping was heat (*n*=10). Besides heat, noise [4], a hard floor [3], a small space [2], and collisions [2] were observed with none of the children claiming anxiety.

## Discussion

In agreement with the results of our previous study [5], sleep was disturbed in C compared to that in other conditions, and heat was identified as the most frequent sleep disturbance, as reported by 10 of the 13 participants. The sleep disturbance in C was concentrated during the initial 195 min. One reason for the increased heat stress may be related to the ambient climate in the initial segment of sleep. The ambient temperature was significantly lower in BC than in A1 and A2, while the significant difference between C and BC was limited during the initial 180 min. The ambient temperature in the initial 180 min in C was accompanied by significantly higher ambient RH than that in A1 and A2, which may have increased heat stress. The subjective sensation before sleep may further support this finding. The subjective humid sensation before sleep was significantly wet in C compared to BC and A2. Furthermore, subjective sweat and hot sensations before sleep were significantly higher in C than in BC. Considering that 85% of the participants did not sleep inside the sleeping bag at sleep onset, it is possible that the temperature and RH during the initial sleep segment may have been perceived by the children as hot. The hot sensation may also be related to cramping up and collisions, and no air flow due to thick curtains on the windows in the gymnasium, leading to higher ambient temperature and humidity in the areas near each participant, as suggested in our previous study [5]. Another factor that should be considered is that the outside temperature during the day and night increased abruptly from BC to C. Although the ambient temperature in C was higher than 26°C in the initial 180 min, it gradually decreased to 25°C which was significantly lower than that in A1 and A2 in the last sleep segment. We did not observe this ambient temperature under other conditions, which suggests that the ambient temperature inside the gymnasium might be more affected by the outside temperature. One reason for this change could be the construction of the gymnasium in which the floor bottom is usually outside in the open air, whereas heat insulators generally exist in homes. One possibility for the absence of a significant difference in sleep time after 195 min might be related to the decreased ambient temperature, since it started to decrease below 26 °C from 180 min. However, decreased sleep time during the initial 195 min may include not only ambient climate but other factors such as the first night effect, the effect of noise, and hard floors, as mentioned in our previous study [5]. Further, no significant difference after 195 min may also include factors besides ambient climates such as increased sleep pressure and decreased noise level [5]. These factors should be considered and need caution when considering the relationship between ambient temperature and sleep in C. However, considering that controlling the ambient temperature in the gymnasium by measuring ambient temperature near each participant is difficult, our study at least indicates the possibility that ambient temperature should be controlled lower than 26 °C during the initial segment of sleep when more than 170 people are staying in the gymnasium.

The disturbed sleep in C was related to a significantly delayed increase in the bed climate temperature in the chest area in C compared to other conditions during the initial 15 to 30 min. One possible explanation for this outcome may be related to the fact that children did not sleep inside the sleeping bag, resulting in low bed climate temperature in the chest area. Past studies on awake subjects imply that the sweating rate is lower in children than in adults [15, 16]. However, children use distinct mechanisms for thermoregulation in hot environments because of greater cutaneous vasodilation of the head and trunk and a greater surface area-to-mass ratio [15, 16]. This suggests that children rely more on dry heat loss than evaporative cooling. Although behavioral thermoregulation of not entering the sleeping bag and sleeping directly on the underside mattress may have increased dry heat loss, it might not have been sufficient to overcome heat stress in C. A previous study on adults found that the covered area of the body, by bed covering during sleep, differs depending on ambient temperature, which is more sensitive in the upper extremities than in the lower extremities or trunk, and the lower extremities are more sensitive than the trunk [17]. This suggests that behavioral thermoregulation is active during sleep. A previous study indicated that behavioral thermoregulation during sleep might be more important in children than in adults [18]. In sleeping bags, behavioral thermoregulation is limited to covering the trunk and lower extremities, or not covering both. Furthermore, staying inside a sleeping bag may limit body movement, sleep position changes, and ventilation inside the bed climate. This constraint may further increase the thermal stress and explain the behavior in C. Considering that the ambient temperatures in the gymnasium can be influenced by outside temperatures and that sleeping bags may hinder behavioral thermoregulation and dry heat loss, different kinds of bedding should be provided to the children in the gymnasium.

Another possibility may be related to disturbed sleep resulting in thermoregulation changes during sleep onset. During the normal sleep onset period in adults, chest and foot skin temperatures and bed climate temperatures increase and are maintained throughout the night [18]. Increased skin temperature (Tsk) and bed climate temperature during the sleep onset period have also been observed in school-aged children [18, 19] and preschool children with normal sleep [10]. Further, previous research and the present study imply that the bed climate temperature increases during the sleep onset period and is maintained throughout the night in school-aged children [10]. However, the Tsk increase during sleep onset may differ between adults and preschool children. Preschool children may rely on chest Tsk for heat dissipation at sleep onset, followed by increases in foot Tsk [10]. In school-aged children, chest Tsk also increases to its maximum level earlier than foot Tsk at sleep onset. Further, foot Tsk at sleep onset takes 90 min to reach the plateau level [19]. This might explain why the significantly delayed temperature increase in bed climate temperature in C compared to other conditions was shorter in the foot area than in chest area. Children’s cutaneous vasodilation of the head and trunk increases in hot environments during a waking state [15, 16]. Thus, increasing proximal Tsk may also be important for school-aged children during sleep onset to decrease cardiovascular strain during sleep [10]. Since the sleep onset period was excessively delayed in C compared to that in the other conditions, the chest Tsk may not have increased at sleep onset, resulting in a lower bed climate temperature of the chest at the initial 15 to 30 min. Considering that sweat sensation before sleep was higher in C than in BC and bed climate humidity of the chest area at initial 5 to 10 min was significantly higher in C than in BC and A1, sweating may have decreased bed climate increase in C.

Although sleep time was lower in C than in other conditions during the initial 195 min, a significant difference in bed climate temperature of the chest area between C and the other conditions was limited to the initial 30 min. This might indicate that regardless of sleep or wakefulness, the bed climate temperature of the chest area does not differ after the sleep onset period. This result might be attributed to the Tsk change during sleep in children. The chest Tsk increases to its maximum at sleep onset and gradually decreases until the timing when the foot Tsk reaches a plateau [19]. The bed climate temperature of the chest area in A1 and A2 also decreased after maximum, and BC maintained a plateau, while it continued to increase in C, leading to no significant difference among the conditions. The Tsk increases due to increased skin blood flow, which has two pathways, one is the noradrenergic vasoconstrictor system, and the other is the active vasodilator system. The Tsk increase at sleep onset is attributed primarily to reduced activation of noradrenergic vasoconstrictor tone [20], while increased Tsk under heat stress during wake is due largely to an increased active vasodilator system [21]. Although we cannot distinguish these two pathways, the bed climate temperature increase in the chest area in C might largely be related to the Tsk increase due to the active vasodilator system, at least during the initial 195 min. Behavioral thermoregulation in C may have also accounted for no significant difference in bed climate temperature of the chest area among the conditions. Although 85% of the children were not inside their sleeping bags, 50% were sleeping inside their sleeping bags upon waking. Since body temperature generally decreases during sleep, ambient temperature decreases in C until 25°C may have been perceived as cool by some children in the morning, leading to increased covering behavior and bed climate temperatures in the later sleep segment. Since children rely on dry heat loss, covering and uncovering behaviors in relation to sleeping bags may have supported homeothermy during later segment of sleep in C.

Time is needed to improve Japanese shelters, as it requires revising both the political system itself and the law. However, the outside temperature is increasing year by year [22]; for example, during summer in 2023, the maximum lowest outside temperature was 27.9°C, the maximum temperature was 39.8°C, and the mean RH was 75%, in Osaka, which is one of the hottest prefectures in Japan [14]. Our results indicate the urgent need to at least set up portable air conditioning in gymnasiums and preparing a system to supply portable beds, mattresses, and bed coverings throughout Japan.

This study has several limitations that should be considered. First, none of our participants claimed anxiety as a sleep disturbance factor since this was an enjoyable event. This signals that mental stress is completely different from real situations involving natural disasters. Second, the number of evacuees in a shelter is generally much greater than in this study, and the effect may increase further. Third, questionnaires about how they used the sleeping bag before and during sleep were recalled after sleep. This might lack accuracy, since the answers are limited to the memories when participants were awake. Fourth, the age and number of participants were limited in this study. Larger sample sizes and different age groups should be considered for future research. Fifth, the results of this study are unsuitable for generalizing because the ambient temperature and humidity inside the gymnasium can be influenced by outside temperatures and the number of people present.

## Conclusions

In summary, these outcomes reveal that a noticeable delay in bed climate temperature increase in the chest area during the sleep onset period may be associated with sleep disturbances in children sleeping in a simulated shelter. Setting up a portable air conditioning in the gymnasium and preparing a system to supply portable beds, mattresses, and bed coverings are necessary measures that should be taken to avoid sleep disturbances in children as well as all the evacuees during future disasters.

## Data Availability

The datasets during and/or analyzed during the current study are available from the corresponding author on reasonable request.

## Abbreviations

RH: Relative humidity
BC: Night before school camp
C: School camp
Tsk: Skin temperature
A1: One day after school camp
A2: Two days after school camp
